# The complete chloroplast genome of *Cucumis anguria var. anguria* (*Cucurbitaceae*) and its phylogenetic implication

**DOI:** 10.1080/23802359.2019.1711231

**Published:** 2020-01-16

**Authors:** Hong Cheng, Wei-ping Kong, Min-min Zhang, Dong Hou

**Affiliations:** a Gansu Academy of Agricultural Sciences, Vegetable Research Institute, Lanzhou, China;; b Key Laboratory of Fruit Breeding Technology, Ministry of Agriculture and Rural Affairs, Zhengzhou, China;; c Gansu Academy of Agricultural Sciences, Institute of Biotechnology, Lanzhou, China

**Keywords:** *Cucumis anguria var. anguria*, complete chloroplast genome, phylogenetic analysis, Cucurbitaceae

## Abstract

The genus *Cucumis* contains 52 species, including two economically significant crops, cucumber and melon, as well as other important species. *Cucumis anguria var. anguria* is a wild relative of *C. melon*, native to Africa. *Cucumis anguria* is rich in vitamins and minerals in gherkin fruits and carries broad-spectrum resistance to multiplex biotic and abiotic stress, such as powdery mildew, fusarium wilt, and meloidogyn incognita. *Cucumis anguria* provides a valuable gene pool for crop improvement of *Cucumis* crops. In this study, the complete chloroplast (cp) genome sequence of *C. anguria* was determined using next-generation sequencing. The entire cp genome was determined to be 156,577 bp in length. It contained large single-copy (LSC) and small single-copy (SSC) regions of 85,971 and 18,100 bp, respectively, which were separated by a pair of 26,253 bp inverted repeat (IR) regions. The genome contained 134 genes, including 88 protein-coding genes, 37 tRNA genes, 8 rRNA genes and 1 pseudogene infA. The overall GC content of the genome is 37.0%. A phylogenetic tree reconstructed by 48 chloroplast genomes reveals that *C. anguria* is a separate branch in *Cucumis*.

The genus *Cucumis* contains 52 species, including two economically significant crops, cucumber and melon, as well as other important species. *Cucumis anguria var. anguria* is a wild relative of *C. melon*, native to Africa **(**Kerje and Grum 2000**)**. *Cucumis anguria* is rich in vitamins and minerals in gherkin fruits and carries broad-spectrum resistance to multiplex biotic and abiotic stress, such as powdery mildew, fusarium wilt, and meloidogyn incognita. *Cucumis anguria* provides a valuable gene pool for crop improvement of *Cucumis* crops. However, genetic relationships between *C. anguria* and other melon species on genomes level have not been studied. So, it is necessary to develop genomic resources for *C. anguria* to provide basic intragenic information for further study on phylogeny and breeding for genus *Cucumis.*

The total genomic DNA was extracted from the fresh leaves of *C. anguria* (Gaolan County, Gansu, China, 36.6 N, 103.38E) using the DNeasy Plant Mini Kit (Qiagen, Valencia, CA, USA). The voucher specimen was deposited at Gansu Academy of Agricultural Sciences (2016Y25). The whole genome sequencing was conducted by Genepioneer Biotechnologies Inc. (Nanjing, China) on the Illumina Hiseq 4000 Sequencing System (Illumina, Hayward, CA, USA). The filtered sequences were assembled using the SPAdes assembler 3.10.0 **(**Bankevich et al. 2012). Annotation was performed using the DOGMA (Wyman et al. 2004). All the tRNA sequences were confirmed using the web-based online tool, tRNAScan-SE (Schattner et al. 2005) with default settings to corroborate tRNA.

The plastome of *C. anguria* was determined to comprise double-stranded, circular DNA of 156,577 bp containing two inverted repeat (IR) regions of 26,253 bp each, separated by large single-copy (LSC) and small single-copy (SSC) regions of 85,971 and 18,100 bp, respectively (NCBI acc. no. MN807280). The genome contained 134 genes, including 88 protein-coding genes, 37 tRNA genes, 8 rRNA genes and 1 pseudogene infA. The eight protein-coding genes, seven tRNA genes, and four rRNA genes were duplicated in the IR region. Seventeen genes contained two exons and four genes (clpP and ycf3 and two rps12) contained three exons. The overall GC content of *C. anguria* cp genome is 37.0% and the corresponding values in LSC, SSC, and IR regions are 34.7, 31.7, and 42.6%, respectively.

To investigate its taxonomic status, whole chloroplast genomes from 21 *Cucumis* plants and two outgroup plants (*Coccinia grandis* and *Carica papaya*) were aligned by MAFFT version 7 (Katoh and Standley 2013). A maximum likelihood (ML) was reconstructed based on FastTree version 2.1.10 (Price et al. 2010). The ML phylogenetic tree shows that *Cucumis anguria* is a separate branch in *Cucumis*, with bootstrap support values of 100% (Figure 1).

**Figure 1.**
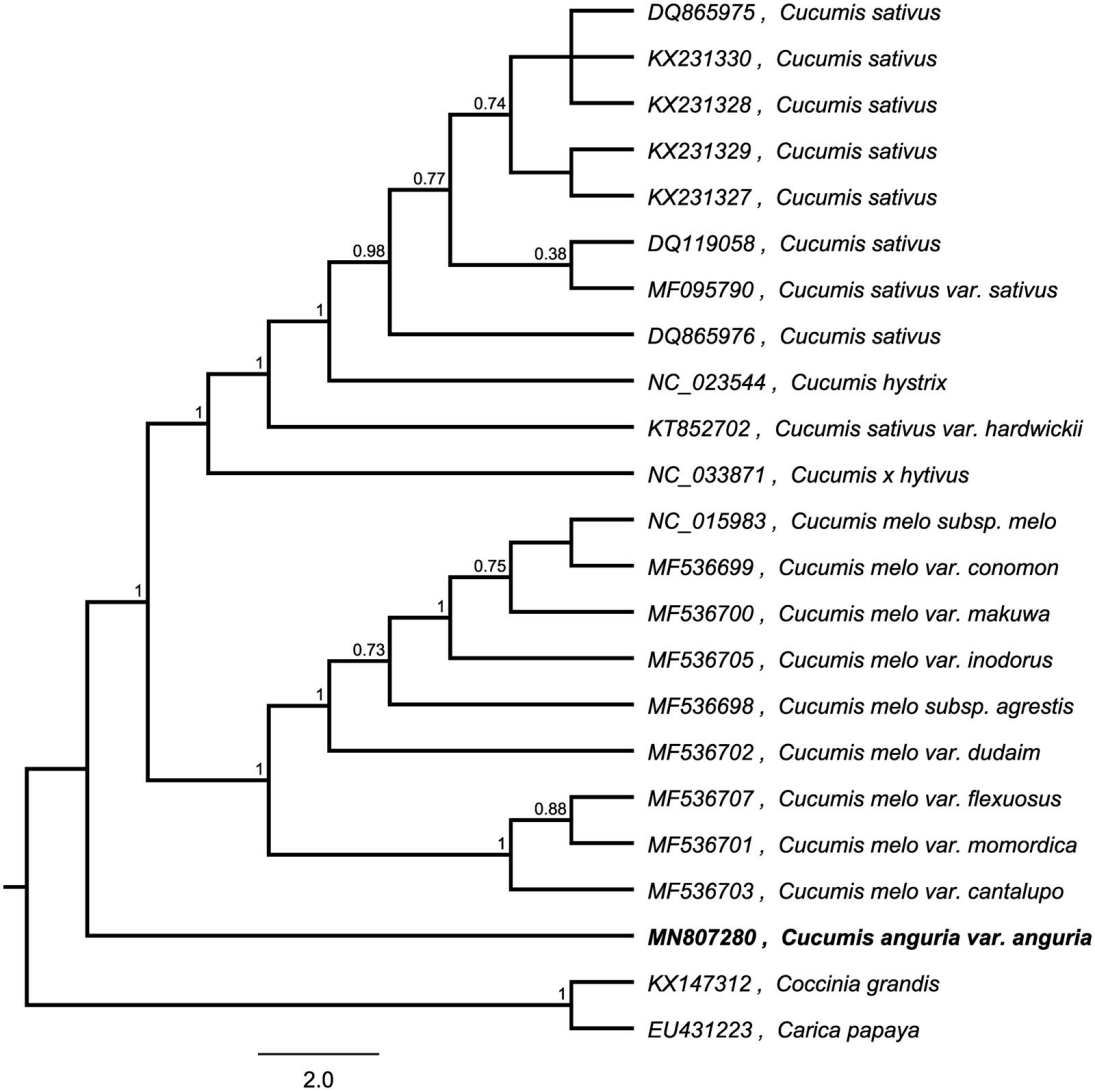
Maximum-likelihood phylogenetic tree for *Cucumis anguria* based on whole chloroplast genomes from 21 *Cucumis* plants and two outgroup plant (*Coccinia grandis* and *Carica papaya*) and the support values are shown at the branches.
